# Supplementary material for: A fault-intrusion-tolerant system and deadline-aware algorithm for scheduling scientific workflow in the cloud
Source: PeerJ Comput Sci. 2021 Nov 2;7:e747. doi: 10.7717/peerj-cs.747 (PMC8576554; doi:10.7717/peerj-cs.747)
Supplement: Supplemental Information 6 [file peerj-cs-07-747-s006.zip › Myproject1.1/docs/org/workflowsim/utils/package-frame.html]

org.workflowsim.utils


# org.workflowsim.utils

## Classes

- ArgumentParser
- ClusteringParameters
- OverheadParameters
- Parameters
- ReplicaCatalog

## Enums

- ClusteringParameters.ClusteringMethod
- Parameters.FTCFailure
- Parameters.FTCMethod
- Parameters.FTCMonitor
- Parameters.PLNMethod
- Parameters.SCHMethod
- ReplicaCatalog.FileSystem
